# Does Autism Diagnosis Age or Symptom Severity Differ Among Children According to Whether Assisted Reproductive Technology was Used to Achieve Pregnancy?

**DOI:** 10.1007/s10803-015-2462-1

**Published:** 2015-05-22

**Authors:** Laura A. Schieve, Christine Fountain, Sheree L. Boulet, Marshalyn Yeargin-Allsopp, Dmitry M. Kissin, Denise J. Jamieson, Catherine Rice, Peter Bearman

**Affiliations:** National Center on Birth Defects and Developmental Disabilities, Centers for Disease Control and Prevention, MS E-86, 1600 Clifton Road, Atlanta, GA 30333 USA; Department of Sociology and Anthropology, Fordham University, New York, NY USA; National Center for Chronic Disease Prevention and Health Promotion, Centers for Disease Control and Prevention, Atlanta, GA USA; Interdisciplinary Center for Innovative Theory and Empirics (INCITE), Columbia University, New York, NY USA

**Keywords:** Infantile autism, Symptom severity, Diagnosis age, Assisted reproductive technology

## Abstract

Previous studies report associations between conception with assisted reproductive technology (ART) and autism. Whether these associations reflect an ascertainment or biologic effect is undetermined. We assessed diagnosis age and initial autism symptom severity among >30,000 children with autism from a linkage study of California Department of Developmental Services records, birth records, and the National ART Surveillance System. Median diagnosis age and symptom severity levels were significantly lower for ART-conceived than non-ART-conceived children. After adjustment for differences in the socio-demographic profiles of the two groups, the diagnosis age differentials were greatly attenuated and there were no differences in autism symptomatology. Thus, ascertainment issues related to SES, not ART per se, are likely the driving influence of the differences we initially observed.

## Introduction

Both assisted reproductive technology (ART) and autism spectrum disorder (ASD) have increased dramatically in past decades (Autism and Developmental Monitoring Network Surveillance Year 2008 Principal Investigators 2012; Blumberg et al. 2013; Baron-Cohen et al. 2009; Roelfsema et al. 2012; Schieve et al. 2012b; CDC et al. 2012). Current estimates of ASD prevalence among US children are between 1 and 2 %, with studies indicating a greater than 70 % increase in just the past decade (Autism and Developmental Monitoring Network Surveillance Year 2008 Principal Investigators 2012; Autism and Developmental Monitoring Network Surveillance Year 2010 Principal Investigators 2014; Blumberg et al. 2013). The annual number of ART births in the US has tripled between 1996 and 2011; the most recent annual estimate is over 60,000 births, approximately 1.5 % of the 2011 US birth cohort (Sunderam et al. 2014). ART is defined in the US and many other national registries as inclusive of only the most intensive infertility treatments, such as in vitro fertilization, in which both sperm and eggs are handled outside of the body.

Several large population-based studies from various countries document that overall, the prevalence of autism or ASD diagnoses is moderately higher among children conceived with ART than among children in the general population (Hvidtjorn et al. 2009, 2011; Sandin et al. 2013; Fountain et al. 2015); however, associations were generally reduced after control of socio-demographic and perinatal factors, such as multiple birth. We recently assessed this association in a large US population-based cohort (Fountain et al. 2015) and observed that the ART-autism association was attenuated after (1) adjustment for socio-demographic factors, such as maternal education and race, likely related to parents’ awareness of ASD and access to and ability to navigate the healthcare system and (2) adjustment for several potential mediating factors—multiple birth, preterm birth (PTB), fetal growth restriction and maternal complications. Our previous study along with other population-based assessments suggests that while ART is associated with ASD, it likely only has a modest, if any, direct effect on ASD etiology.

In the current study, we further explore whether and to what extent the ART-autism association is specifically driven by differences in autism identification patterns between ART- and non-ART-conceived children. We assessed child’s age and symptom severity at autism identification. Trends toward both earlier identification and increased identification of children with less “severe” symptom profiles across all ages have been shown to be notable contributors to the increased US prevalence of identified ASD overall (Shattuck et al. 2009; Autism and Developmental Monitoring Network Surveillance Year 2006 Principal Investigators 2009; Autism and Developmental Monitoring Network Surveillance Year 2008 Principal Investigators 2012; Schieve et al. 2012b; Blumberg et al. 2013). We assessed whether these dynamics were especially pronounced among children conceived with ART. For several reasons, parents and healthcare providers might monitor ART-conceived children more closely than other children for health and developmental difficulties. ART-conceived children are typically from families with higher than average socioeconomic status and thus better than average access to healthcare (Schieve et al. 2007); their parents have experience navigating the complex health care system for infertility treatment, and this could certainly translate to being more savvy in navigating the pediatric care system; ART-conceived children are more likely than non-ART-conceived children to have an adverse perinatal outcome, such as PTB or low birth weight (Farhi et al. 2013; Schieve et al. 2007); and ART mothers might be more likely to be concerned about their baby’s health generally (Barnes et al. 2012).

We assessed diagnosis age and levels of initial social and communication deficits for California children born between 1997 and 2006 who were subsequently diagnosed with autism. We compared children conceived with ART to those not conceived with ART. Additionally, we assessed various subgroups of the population and controlled for socio-demographic factors and adverse perinatal outcomes to better understand the reasons for any differences observed between ART- and non-ART-conceived children.

## Methods

### Data Sources

We used data from a previous linkage of three large population-based datasets: the California Birth Master Files (BMF) for 1997–2007, the California Department of Developmental Services (DDS) autism caseload records for 1997–2011 (an administrative database known as CDER, client development evaluation reports), and the Centers for Disease Control and Prevention’s (CDC’s) National ART Surveillance System (NASS).

The California DDS is a statewide agency responsible for coordinating diagnoses and services for persons with developmental disabilities including autism. Children are referred to DDS regional centers from health-care providers, educators, service agencies, public health clinics, and parents. To quality for services, a child must have a certain level of functional deficit. While children with Autistic Disorder (DSM-IV code 299.0) have generally qualified, those with other ASDs, generally have not. Thus, these data represent a subset of children with ASD—those likely to have more significant functional limitations. We thus use the terminology autism throughout this report to describe our study population, rather than the broader term, ASD. Although autism cases included in the DDS are not identified through systematic population surveillance, eligibility for services is based on diagnostic and not financial criteria, and thus, the system captures the vast majority of the population. Even so, it is possible that certain population subgroups are over-represented, such as children from families with socioeconomic advantages who are able to navigate the complex system more easily. CDER is one of the largest administrative sources of data on autism diagnoses in the US Moreover, a previous comparison of a random sample of CDER autism cases with medical record data indicates high reliability (Croen et al. 2002a).

NASS includes data on women who receive ART services from healthcare providers in the United States and its territories. US clinics and medical practices are federally mandated to annually report data to the CDC for every ART procedure initiated (United States 1992). While 5–10 % of clinics do not report as mandated, many non-reporting clinics are thought to be smaller than average practices because they are either new practices or practices in process of reorganizing or closing (CDC et al. 2012). Data in NASS are abstracted by clinic personnel from patient records; in addition to clinical information on each ART treatment, data are abstracted on resultant pregnancies and pregnancy outcomes. Pregnancy outcome data are obtained for 99 % of all ART pregnancies, often through active follow-up. Annual on-site data validation visits at a sample of reporting clinics have consistently confirmed the accuracy of pregnancy and birth reporting (CDC et al. 2012).

### Linkage Procedures

The linkage procedure has been previously described (Zhang et al. 2012). Briefly, we selected from NASS the subset of ART procedures that were performed on in-state residents in California clinics/medical practices and resulted in a live birth. These data were linked to the BMF based on mother’s date of birth, infant’s date of birth, plurality, mother’s ZIP code, and gravidity. Uncertain matches were manually reviewed, and infant sex, maternal race, and infant birth weight were used to resolve duplicate or uncertain matches. Ninety percent of the ART births selected from NASS for this study were successfully linked to a California birth from BMF.

Autism cases from CDER were also linked to BMF probabilistically on first and last names, middle initial, date of birth, sex, race/ethnicity, and maternal zip code. Uncertain matches were manually reviewed. On average, 86 % of eligible children with autism in CDER were linked to a birth record. This linkage rate is in line with data from a previous linkage study of the same two datasets (Croen et al. 2002a). Typically, CDER data that could not be matched belonged to children born outside of California who had moved into the state at some time after their births.

This protocol was approved by the Institutional Review Boards at Columbia University and the Centers for Disease Control and Prevention, and by the California Committee for the Protection of Human Subjects.

### Study Population

**Fig. 1:**
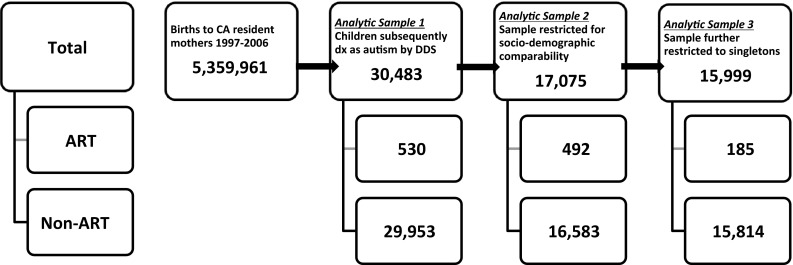
Sample selection process and sample sizes according to whether a child was conceived with ART

Our sample selection is illustrated in Fig. 1. Between 1997 and 2006, there were 5,359,961 children born to California resident mothers. In our initial analyses, we selected from this population, 30,483 children who were subsequently diagnosed with autism through the DDS system; 530 of these children were conceived with ART.

We conducted a series of analyses using 3 samples. Our initial sample consisted of the total population of 30,483 children with autism. Our second sample was constructed from this initial sample. We restricted the sample to account for the marked differential in the socio-demographic profile for ART versus non-ART births. We thus excluded children whose mothers were less than 20 years of age at the time of their birth, had less than a high school diploma, had prenatal care or delivery paid for by Medi-Cal or other public source, or had missing information on prenatal care, inadequate prenatal care (Kotelchuck 1994) or started prenatal care in the third trimester. Additionally, we excluded infants with missing values for other socio-demographic factors that were included as covariates in our statistical models (about 4 %, of the sample). Within each of the aforementioned population subgroups excluded from our second sample, the proportion of ART births was ≤3.0 %; thus, sample restriction was preferable to solely using statistical adjustment to account for potential confounding factors (i.e. the likelihood of residual confounding was high for statistical adjustment alone). This sample restricted for socio-demographic comparability (henceforth referred to as the restricted sample) included 17,075 infants of whom 492 were conceived with ART. It is notable that while nearly half of the non-ART births in our initial sample were excluded from our restricted sample, only 7 % of ART births were excluded. This highlights our rationale for sample restriction. In our final analytic sample, we additionally excluded children who were born in a multiple-birth delivery and children missing gestational age and birthweight data. Multiple births were excluded from this sample to assess effects in the absence of potential perinatal mediators; multiple births are strongly associated with the use of ART and convey a much higher risk for adverse maternal and infant health outcomes and child disability than singleton births (Schieve 2007). This sample, henceforth referred to as the restricted singleton sample, included 15,999 children of whom 185 were conceived with ART.

For certain analyses—those assessing communication level, and social functioning at initial DDS assessment—each of our analytic samples was further reduced. Because of changes in the rating items that comprised these two measures (see below), we were only able to include children born between 1997 and 2004 in those analyses.

### Outcomes

Our outcomes of interest -age at autism diagnosis, autism communication and social functioning severity indicators, and co-occurring ID—were derived from CDER data. Diagnosis age was calculated from date of birth and date of first DDS evaluation. California requires that all children receiving DDS services are confirmed as meeting eligibility for services through verification or confirmation of a diagnosis within 120 days of intake; thus, the first DDS visit date is considered to be very close to diagnosis date. Because children with developmental delays who are younger than 3 years of age are served by the Early Start Program, age at diagnosis in those younger than three is rarely found in the DDS records. We assessed both mean and median age at diagnosis as well as percentage of children with early diagnosis, defined here as <4 years of age.

At a child’s first DDS evaluation, communication and social functioning are systematically assessed via ratings for a series of Likert-scale items, and these ratings are recorded in the CDER. Communication items include: word usage; receptive language; and expressive language. Social functioning items include: level of social interaction with peers; level of social interaction with non-peers; friendship formation and maintenance; and participation in social activities. We created indices for communication and social functioning by combining scores for all items for a given domain; for each index, individual items within the domain were weighted equally (Fountain et al. 2012). The specific items used to assess both communication and social functioning changed in 2008 and thus, the children included in our study population were assessed using two different (albeit related) metrics. Children born between 1997 and 2004 were primarily assessed with the pre-2008 criteria, while children born in 2005–2006 were assessed with the newer criteria. We attempted to harmonize the two indices into a common set of criteria for analyses. We used two approaches—one based on empirical assessments of item frequencies and one based on a priori expert clinical judgment. Neither process produced satisfactory results; thus, we limit our assessment of the communication and social functioning outcomes to children born between 1997 and 2004 who were assessed using the pre-2008 criteria (N = 19,518 for the initial sample). For these children, we first assessed the overall distributions of the calculated communication and social index scores. We defined low functioning for each index as having a score in the bottom tertile.

In addition to the functional indices for core autism symptomatology, we also assessed the percentage of children with a co-occurring diagnosis of ID (IQ <70), an indicator related to one aspect of condition severity. We lacked access to IQ data to further refine this indicator to assess various levels of intellectual functioning among children with and without ID. Additionally, it is likely that for a proportion of children with autism who had co-occurring ID, the ID classification was not included in the CDER database. An early reliability study based on medical record review indicated that while autism was reliably reported in CDER, there was a noteworthy level of under-reporting of co-occurring ID among children with autism (Croen et al. 2002b). Also, because CDER is an administrative rather than a research database, children are not necessarily systematically assessed for all developmental conditions. The focus is on service provision, and thus, a child with autism might not be assessed for all secondary conditions if services being provided for the primary condition will also cover his/her secondary functional deficits. Even though we believe ID was underreported, we have no reason to believe the level of under-reporting varied by mode of conception. Thus, we included it in our analyses as an adjunct severity indicator.

### ART

ART was defined to include nearly all types of ART reported to NASS: treatments in which freshly-fertilized embryos created using the intended mother’s own eggs were transferred; treatments in which freshly-fertilized embryos created using another woman’s (donor) eggs were transferred; treatments in which previously frozen, thawed embryos created using either the intended mother’s or donor eggs were transferred; treatments using standard in vitro fertilization techniques as well as treatments using intracytoplasmic sperm injection (ICSI), and treatments using the more standard trans-cervical embryo transfer technique as well as those using gamete or zygote intrafallopian transfer (GIFT, ZIFT). However, ART in this analysis does not include the very small percentage of procedures in which embryos that were created using an ART were transferred into a woman other than the intended mother (a gestational surrogate), because these types of procedures were excluded from the NASS-birth certificate linkage. Also, NASS defines ART as including only those procedures in which egg and sperm are handled outside the body; thus infertility treatments such as ovulation stimulation only without egg retrieval and artificial insemination are not collected in NASS.

### Covariates and Causal Path Factors

In all adjusted models we included child sex, maternal age at child’s birth, maternal educational level at child’s birth, maternal race-ethnicity and maternal immigration status (US versus foreign-born) as potential confounders. In our final models, we additionally included two factors potentially in the causal pathway for the ART-ASD association, PTB and small-for-gestational-age (SGA). Adverse perinatal outcomes have been found to be associated with ART use previously, even when considering singleton deliveries only (Schieve et al. 2007). All covariates were derived from birth certificate data. PTB was defined as gestational age <37 completed weeks (based on last menstrual period or clinical estimate when missing). SGA was defined as birthweight-for-gestational age <10th ‰ of a US referent population (Oken et al. 2003).

### Statistical Analysis

We evaluated all diagnosis age outcomes and co-occurring ID in four sets of analyses: (1) unadjusted analyses of our total study population of children diagnosed with autism; (2) analyses of the restriction sample with additional adjustment for demographic factors; (3) analyses of restricted, singleton sample with additional adjustment for demographic factors; (4) analyses of restricted, singleton sample with additional adjustment for both demographic and causal path factors. We evaluated communication and social functioning outcomes in the first two analyses sets only; sample sizes were insufficient to include these outcomes in the third and fourth sets.

For all dichotomous outcomes, we calculated odds ratios (ORs) and 95 % confidence intervals in which the odds of the outcome among ART-conceived children were compared to the odds for non-ART-conceived children. Adjusted ORs were computed using logistic regression.

We also assessed the mean and median diagnosis age for ART- versus non-ART-conceived children and calculated the mean differences using linear regression models with log-transformed age values to account for skewed data. We included the same adjustment factors in these models as for dichotomous outcomes in analyses sets 2 through 4. We used SAS statistical software version 9.3 (SAS Institute) to conduct all analyses.

All analyses were conducted within birth year strata (1997–1999; 2000–2002; 2003–2004; 2005–2006) as well as for the total sample. Because both ART and autism have increased over time, it is feasible that diagnosis age and autism severity level at initial assessment have changed as well.

## Results

In all three of our analytic samples, ART-conceived children were more likely than non-ART conceived children to be female and to be from more recent birth cohorts (Table 1). In all three samples, mothers of ART-conceived children were more likely than mothers of non-ART—conceived children to be non-Hispanic white (NHW), and they were less likely to be Hispanic and born outside the US. Additionally at the time of the child’s birth, ART mothers were substantially more likely than non-ART mothers to be primiparous, older, to have completed four or more years of college, and to have received greater than adequate prenatal care. In the total and the restriction samples, >60 % of ART-conceived children were from multiple births versus <5 % among non-ART-conceived children and thus, ART-conceived children were also much more likely to be born PTB and SGA. However, even in the restricted, singleton sample, ART-conceived children were more likely than non-ART-conceived children to have PTB and SGA.

**Table 1:** Percentage distributions of socio-demographic and perinatal factors by ART status in the total study population of children with autism and analytic subsamples

	Total population of children with autism		Sample restricted for socio-demographic comparability		Sample further restricted to singletons	
	ART births (N = 530)	Non-ART births (N = 29,953)	ART births (N = 492)	Non-ART births (N = 16,583)	ART births (N = 185)	Non-ART births (N = 15,814)
Year of birth						
1997–1999	21.1	25.1	20.5	26.0	21.1	26.3
2000–2002	31.1	31.2	31.5	32.5	28.1	32.7
2003–2004	22.1	23.2	22.2	22.6	20.0	22.5
2005–2006	25.7	20.5	25.8	18.9	30.8	18.6
Child sex						
Female	20.2	16.8	19.5	16.8	18.4	16.7
Male	79.8	83.2	80.5	83.2	81.6	83.3
Maternal race/ethnicity						
Non-Hispanic white	66.8	34.0	68.1	45.8	69.2	45.6
Non-Hispanic black	4.2	7.5	3.9	6.6	4.9	6.0
Hispanic	12.3	41.1	11.4	24.9	7.0	24.9
Asian/Pacific Islander	14.9	15.9	14.8	21.2	17.8	21.4
Other/unknown	1.9	1.5	3.4	1.5	–^a^	1.5
Maternal birthplace						
US and territories	71.3	56.1	72.2	63.6	71.4	63.2
Outside US	28.7	43.9	27.9	36.4	28.7	36.8
Maternal education at child’s birth						
<high school	1.4	19.1	–	–	–	–
High school	8.2	26.6	7.4	22.9	4.0	23.0
Some college	21.3	24.7	21.9	30.2	17.0	30.5
4-year college grad +	69.1	29.6	70.8	46.9	79.1	46.5
Maternal age (y) at child’s birth						
<20	0	5.2	–	–	–	–
20–24	–	18.1	0.6	10.6	–	10.8
25–29	5.3	25.8	4.9	25.4	3.8	25.7
30–34	25.1	28.2	24.8	34.9	18.9	34.8
35–39	33.8	18.1	33.7	23.2	36.2	22.8
40+	35.3	4.7	36.0	5.9	40.0	5.8
Parity at child’s birth						
Primiparous	55.1	43.2	55.7	46.7	82.7	47.8
Multiparous	44.9	56.8	44.3	53.3	17.3	52.2
Prenatal care initiation						
First trimester	98.3	88.9	98.6	96.5	97.8	96.4
Second trimester	1.4	9.2	1.4	3.5	-	3.6
Third trimester	–	1.6	–	–	–	–
No prenatal care	0	0.3	–	–	–	–
Adequacy of prenatal care						
Inadequate	–	6.9	–	–	–	–
Intermediate	2.8	11.5	2.9	12.0	5.4	12.5
Adequate	14.9	40.9	16.1	45.2	30.8	46.7
Adequate +	78.9	38.4	81.1	42.8	63.8	40.7
Missing	2.8	2.4	–	–	–	–
Payment source prenatal care						
Private insurance/payment	97.1	64.4	100	100	100	100
Public source	2.9	35.6	–	–	–	–
Payment source delivery						
Private insurance/payment	97.1	64.1	100	100	100	100
Public source	2.9	35.9	–	–	–	–
Birth plurality						
Singleton	37.4	95.9	37.6	95.4	100	100
Twin	50.9	3.9	50.4	4.4	–	–
Triplet/+	11.7	0.2	12.0	0.2	–	–
Preterm birth						
Yes	47.1	12.7	47.8	12.1	18.3	9.9
No	52.9	87.3	52.2	87.9	81.7	90.1
Small-for-gestational-age						
Yes	27.8	10.5	28.8	10.2	13.1	9.1
No	72.2	89.5	71.2	89.8	86.9	90.9

The sample restricted for socio-demographic comparability excludes children whose mothers were less than 20 years of age at the time of their birth, had less than a high school diploma, had prenatal care or delivery paid for by Medi-Cal or other public source, or had missing information on prenatal care, inadequate prenatal care or started prenatal care in the third trimester. The sample further restricted to singletons excludes all of the aforementioned children and additionally excludes all children born in twin or higher-order multiple birth deliveries

Data not presented if number of observations in a given category was < 5

^a^Statistical testing not performed; these data are presented to provide a general sense of differences in socio-demographic characteristics and pregnancy outcomes between study groups within and across analysis samples. These differences were accounted for in subsequent analyses through statistical adjustment and/or sample restriction

Comparisons of ART- and non-ART-conceived children on autism diagnosis age are presented in Tables 2 and 3. We found that for the total study population of children with autism, those conceived with ART had significantly lower mean and median autism diagnosis ages than those not conceived with ART (Table 2). This finding was consistent for all birth cohorts except the most recent (2005–2006). Children conceived via ART and born in 1997–1999 had mean and median diagnosis ages of 4.6 and 3.8 years, respectively. These compare to 5.3 and 4.4 years for the non-ART births during the same time period. In contrast, for the 2005–2006 birth cohorts, mean and median diagnosis ages were notably lower for both ART (3.6 and 3.4 years) and non-ART conceived children (3.7 and 3.5 years). For the total study population, mean diagnosis age was 0.1 year (1.2 months) earlier for ART than non-ART conceived children and this difference was statistically significant (Table 3).

**Table 2:** Diagnosis age outcomes for the total sample and analytic sub-samples by birth year and ART status

Outcome and birth year	Total population of children with autism		Sample restricted for socio-demographic comparability		Sample further restricted to singletons	
	ART births	Non-ART births	ART births	Non-ART births	ART births	Non-ART births
Mean age autism dx (year)						
1997–1999	4.6	5.3	4.7	5.1	4.9	5.1
2000–2002	4.2	4.6	4.2	4.5	4.0	4.5
2003–2004	3.7	4.1	3.6	4.0	3.9	4.0
2005–2006	3.6	3.7	3.7	3.7	3.8	3.7
Total 1997–2006	4.0	4.5	4.0	4.4	4.1	4.4
Median age autism dx (year)						
1997–1999	3.8	4.4	3.8	4.2	4.0	4.2
2000–2002	3.3	4.0	3.2	3.9	3.2	3.9
2003–2004	3.1	3.8	3.1	3.6	3.3	3.6
2005–2006	3.4	3.5	3.6	3.4	3.8	3.4
Total 1997–2006	3.3	4.0	3.3	3.9	3.6	3.9
% diagnosed at <4 years of age						
1997–1999	55.4	40.4	55.5	43.9	51.3	43.9
2000–2002	63.0	48.8	63.9	52.8	69.2	52.5
2003–2004	74.4	55.9	75.2	59.1	64.9	58.7
2005–2006	68.4	65.6	67.7	67.9	66.7	67.4
Total 1997–2006	65.3	51.8	65.7	54.8	63.8	54.4

The sample restricted for socio-demographic comparability excludes children whose mothers were less than 20 years of age at the time of their birth, had less than a high school diploma, had prenatal care or delivery paid for by Medi-Cal or other public source, or had missing information on prenatal care, inadequate prenatal care or started prenatal care in the third trimester. The sample further restricted to singletons excludes all of the aforementioned children and additionally excludes all children born in twin or higher-order multiple birth deliveries

**Table 3:** Measures of association for comparison of ART conceived children to non-ART conceived children for diagnosis age outcomes

Outcome and birth year	Total population no adjustment	Restriction sample + adjustment for socio-demographic factors^a^	Restriction, singleton sample + adjustment for socio-demographic factors	Restriction, singleton sample + adjustment for socio-demographic and causal path factors
*Mean age autism dx (year)*	*Mean difference (log-transformed)*	*Mean difference (log-transformed)*	*Mean difference (log-transformed)*	*Mean difference (log-transformed)*
1997–1999	−0.13*	−0.08	−0.06	−0.10
2000–2002	−0.10*	−0.04	−0.07	−0.07
2003–2004	−0.10*	−0.07*	−0.03	−0.001
2005–2006	−0.02	0.01	0.04	0.04
All birth years	−0.10*	−0.06*	−0.04	−0.05
*Diagnosis at <4 years of age*	*OR (95 % CI)*	*OR (95 % CI)*	*OR (95 % CI)*	*OR (95 % CI)*
1997–1999	**1.8** (**1.3**–**2.7**)	1.4 (0.94–2.1)	1.3 (0.7–2.4)	1.4 (0.7–2.8)
2000–2002	**1.8** (**1.3**–**2.5**)	1.4 (0.95–1.9)	1.7 (0.9–3.0)	1.6 (0.9–3.1)
2003–2004	**2.3** (**1.5**–**3.5**)	**1.7** (**1.1–2.7**)	1.2 (0.6–2.3)	0.9 (0.4–1.9)
2005–2006	1.1 (0.8–1.6)	0.9 (0.6–1.3)	0.8 (0.5–1.5)	0.9 (0.5–1.6)
All birth years	**1.8** (**1.5**–**2.1**)	**1.4** (**1.1**–**1.7**)	1.3 (0.9–1.7)	1.3 (0.9–1.8)

The sample restricted for socio-demographic comparability excludes children whose mothers were less than 20 years of age at the time of their birth, had less than a high school diploma, had prenatal care or delivery paid for by Medi-Cal or other public source, or had missing information on prenatal care, inadequate prenatal care or started prenatal care in the third trimester. The sample further restricted to singletons excludes all of the aforementioned children and additionally excludes all children born in twin or higher-order multiple birth deliveries

Statistically significant values are given in bold

* *p* < 0.05

^a^All adjusted models included child sex, maternal age at child’s birth, maternal educational level at child’s birth, maternal race-ethnicity and immigration status as potential confounders. The final model additionally included PTB and SGA

While mean and median diagnoses ages were very similar for those children retained in the restricted sample as those for children in the total sample (Table 2), after additional adjustment for socio-demographic factors the differential between ART and non-ART children in the restricted sample was greatly reduced and not statistically significant for most birth cohorts (Table 3). Still, for all birth cohorts combined, mean diagnosis age was 0.06 years lower for ART than non-ART children in the restricted sample and this difference was statistically significant. There was even less variation in diagnosis age between ART and non-ART children in the restricted, singleton sample, and there were no statistically significant differences.

We observed the same pattern of results when we assessed diagnosis age as a dichotomous outcome. Among children in the total population sample, the odds of early diagnosis (<4 years) were 80 % higher for ART-conceived than non-ART-conceived children [OR 1.8 (1.6–2.1)] (Table 3). This was reduced in the restriction sample after adjustment for socio-demographic factors [1.4 (1.1–1.7)] and further reduced and no longer significant in the restricted, singleton sample [1.3 (0.9–1.8)]. The addition of the two causal path factors (PTB and SGA) to the model had no additional influence on the findings.

The findings for the three autism severity indicators we examined are presented in Tables 4 and 5. Overall, co-occurring ID was less common among ART-conceived (14.7 %) than non-ART-conceived children (20.3 %) (Table 4), and this association was statistically significant [OR 0.7 (0.5–0.9)] (Table 5). The prevalence of co-occurring ID decreased with each successive birth cohort for both ART- and non-ART-conceived children, but a similar differential between ART- and non-ART-conceived children was observed for each birth cohort. In the restricted sample there was no longer an association between ART and co-occurring ID after adjustment for socio-demographic factors. Nor were associations observed in analyses of the restricted, singleton sample after adjustment for socio-demographic factors only or both socio-demographic and causal path factors.

**Table 4:** Autism severity indicators for the total sample and analytic sub-samples by birth year and ART status

Outcome and birth year	Total population of children with autism		Sample restricted for socio-demographic comparability		Sample further restricted to singletons	
	ART births	Non-ART births	ART births	Non-ART births	ART births	Non-ART births
% diagnosed with co-occurring ID						
1997–1999	22.3	27.0	23.8	24.3	15.4	23.9
2000–2002	17.6	22.2	18.1	18.9	15.4	19.0
2003–2004	11.1	17.4	9.2	13.5	2.7	13.4
2005–2006	8.1	12.7	7.9	10.1	10.5	10.5
Total 1997–2006	14.7	20.3	14.6	17.5	11.4	17.4
% low score (first tertile) communication index at first DDS evaluation						
1997–1999	23.2	32.2	23.5	26.8	–	–
2000–2002	21.3	33.3	21.6	27.6	–	–
2003–2004	17.8	31.0	14.5	25.2	–	–
2005–2006	–	–	–	–	–	–
Total 1997–2004	20.9	32.4	20.3	26.8		
% low score (first tertile) social functioning index at first DDS evaluation						
1997–1999	16.7	32.9	16.3	29.5	–	–
2000–2002	22.7	31.7	23.9	28.6	–	–
2003–2004	25.6	29.2	24.1	25.9	–	–
2005–2006	–	–	–	–	–	–
Total 1997–2004	21.5	31.6	21.6	28.3		

The sample restricted for socio-demographic comparability excludes children whose mothers were less than 20 years of age at the time of their birth, had less than a high school diploma, had prenatal care or delivery paid for by Medi-Cal or other public source, or had missing information on prenatal care, inadequate prenatal care or started prenatal care in the third trimester. The sample further restricted to singletons excludes all of the aforementioned children and additionally excludes all children born in twin or higher-order multiple birth deliveries

Data not presented if number of observations in a given category was <5

**Table 5:** Measures of association for comparison of ART conceived children to non-ART conceived children for autism severity indicators

Outcome and birth year	Total population no adjustment	Restriction sample + adjustment for socio-demographic factors^a^	Restriction, singleton sample + adjustment for socio-demographic factors	Restriction, singleton sample + adjustment for socio-demographic and causal path factors
*Co-occurring ID*	*OR (95 % CI)*	*OR (95 % CI)*	*OR (95 % CI)*	*OR (95 % CI)*
1997–1999	0.8 (0.5–1.2)	1.1 (0.7–1.9)	0.8 (0.3–1.8)	0.8 (0.3–2.0)
2000–2002	0.8 (0.5–1.1)	1.1 (0.7–1.7)	0.8 (0.4–2.0)	1.0 (0.5–2.2)
2003–2004	0.6 (0.3–1.1)	0.8 (0.4–1.5)	–	–
2005–2006	0.6 (0.3–1.1)	1.1 (0.5–2.1)	1.6 (0.7–3.7)	1.3 (0.5–3.4)
All birth years	**0.7** (**0.5**–**0.9**)	1.0 (0.8–1.3)	0.8 (0.5–1.2)	0.8 (0.5–1.3)
*Low score (first tertile) communication index at first DDS evaluation*	*OR (95 % CI)*	*OR (95 % CI)*		
1997–1999	**0.6** (**0.4**–**1.0**)	1.1 (0.7–1.8)		
2000–2002	**0.5** (**0.4**–**0.8**)	0.9 (0.6–1.4)		
2003–2004	**0.5** (**0.3**–**0.8**)	0.6 (0.3–1.1)		
2005–2006	–	–		
All birth years	**0.6** (**0.4**–**0.7**)	0.9 (0.7–1.2)		
*Low score (first tertile) social functioning index at first DDS evaluation*	*OR (95 % CI)*	*OR (95 % CI)*		
1997–1999	**0.4** (**0.3**–**0.7**)	**0.5** (**0.3**–**0.9**)		
2000–2002	**0.6** (**0.4**–**0.9**)	0.9 (0.6–1.4)		
2003–2004	0.8 (0.5–1.3)	1.1 (0.6–1.9)		
2005–2006	–	–		
All birth years	**0.6** (**0.5**–**0.8**)	0.8 (0.6–1.1)		

The sample restricted for socio-demographic comparability excludes children whose mothers were less than 20 years of age at the time of their birth, had less than a high school diploma, had prenatal care or delivery paid for by Medi-Cal or other public source, or had missing information on prenatal care, inadequate prenatal care or started prenatal care in the third trimester. The sample further restricted to singletons excludes all of the aforementioned children and additionally excludes all children born in twin or higher-order multiple birth deliveries

Statistically significant values are given in bold

^a^All adjusted models included child sex, maternal age at child’s birth, maternal educational level at child’s birth, maternal race-ethnicity and immigration status as potential confounders. The final model additionally included PTB and SGA

Although the sample for analyses of communication and social functioning indices was limited to children born in or before 2004 due to changes in evaluation criteria, the findings matched those for co-occurring ID. Children conceived with ART were significantly less likely than those not conceived with ART to present at first evaluation with the most severe deficits in both communication [20.9 vs. 32.4 %, OR 0.6 (0.4–0.7)] and social functioning [21.5 vs. 31.6 % OR 0.6 (0.5–0.8)]. However, neither of these associations was evident in the restricted sample after control of socio-demographic factors.

## Discussion

The findings of this study demonstrate that two possible reasons for the increase in autism observed generally among ART-conceived children are that ART-conceived children have been more likely to receive earlier diagnoses and come to attention of the healthcare system with less severe deficits on average than their non-ART-conceived counterparts. This study provides important context to our previous study of the association between ART and autism (Fountain et al. 2015). In that earlier study, we reported that while some of the ART-autism association in the population overall was accounted for by socio-demographic factors, the association was nonetheless still evident even after restriction based on the same criteria applied here and after control for many socio-demographic confounding factors. However, the association was greatly attenuated after accounting for causal path factors such as multiple birth, SGA, and PTB. The residual association we observed in that earlier study after adjustment for socio-demographic factors might thus be explained by either a biologic effect, i.e. an impact due to ART-conceived children being more likely to have a sub-optimal perinatal environment that directly impacts subsequent neurodevelopment, or an ascertainment effect, i.e. increased developmental monitoring of ART children who are more likely born with one or more adverse perinatal outcomes.

Here we report that the differential in autism diagnosis age was largely accounted for by socio-demographic differences between ART-conceived and non-ART conceived children, and the differentials in autism severity indicators were entirely accounted for by socio-demographic differences. Thus, earlier identification of children with less severe symptomatology who come from more advantaged families appears to be one primary mechanism behind the overall ART-autism association observed in this population overall.

In contrast to our previous study, we found that after adjustment for socio-demographic factors, there was little evidence that either diagnosis age or severity level was different by ART status. That is, additional restriction or adjustment to account for the possible effects from the higher rates of multiple birth and preterm birth among children conceived with ART did not further impact our findings. As mentioned, in our previous study of the overall association between ART and autism we noted residual associations even after adjustment for socio-demographics; these residual associations were largely explained by perinatal factors. While we cannot fully evaluate the mechanism underlying those previously-described findings our current findings for diagnosis age and case severity argue against the hypothesis that adverse birth outcomes simply lead to further enhanced developmental monitoring and increased case-finding in these more vulnerable groups of children who are disproportionately represented in the ART group.

We also found that the diagnosis age differential between ART-conceived and non-ART-conceived children has changed over time. Mean diagnosis ages declined for both groups between 1997–1999 and 2005–2006 such that in the latter birth cohort there was no difference in diagnosis age between the ART and non-ART children. This finding needs to be assessed again in later cohorts when the data become available to determine if the finding is stable. Because we lacked data on two severity indicators for children born in 2005–2006, we could not fully assess time trends. However, the percentage of children with co-occurring ID was lower in ART-conceived compared to non-ART-conceived children in all birth cohorts including 2005–2006.

In the US overall, both trends toward earlier autism identification and increasing identification of children with less severe symptomatology have been documented. Reports from the Autism and Developmental Disabilities Monitoring (ADDM) Network, a population-based surveillance system of ASD in select US sites, show the median age of first ASD diagnosis decreased from 5.7 years for children included in the 2002 surveillance year to 4.4 years by the 2006 surveillance year (Shattuck et al. 2009; Autism and Developmental Monitoring Network Surveillance Year 2006 Principal Investigators et al. 2009). Likewise, data from the National Survey of Children’s Health (NSCH) indicate that while in 2003 ASD prevalence increased gradually with child age, reaching a peak at age 7 years, by 2007 a prevalence peak was observed much earlier, by 5 years of age (Schieve et al. 2012b). ADDM data also indicate that the proportion of children with ASD who have a co-occurring ID has decreased over time (Autism and Developmental Monitoring Network Surveillance Year 2010 Principal Investigators 2014; Autism and Developmental Monitoring Network Surveillance Year 2008 Principal Investigators 2012). Similarly, analyses of NSCH data document that late diagnoses of ASD in children rated by their parents as being on the milder end of the autism spectrum were a major contributor to the recent ASD prevalence increase (Blumberg et al. 2013). Here we find that these two dynamics are also important in studying ASD prevalence variation in population subgroups, such as ART- versus non-ART-conceived children.

While the trend toward decreasing autism diagnosis age that has been observed throughout the population would seem to be the driving force behind the trends we report here for both ART and non-ART children, the convergence of the mean autism diagnosis age in the two study groups that we observed in the latest time period might also be partially attributable to changes in the population of women accessing ART treatments. The prevalence of ART use in California and elsewhere in the US has increased markedly over the time period covered by this study (CDC et al. 2012). While California’s insurance mandate that specified group health plans offer coverage for ART procedures was in place before 1997, the increase in ART use nonetheless demonstrates that more women were able to or choosing to access these treatments in recent periods. Although even in the most recent time periods, the women who conceived via ART remained a highly select group of the total population of women giving birth, the ART trend might nonetheless have influenced the differential between mothers of ART and non-ART children, such that there is less variation between the two groups on pediatric care-seeking behaviors.

This study should be interpreted in the context of several limitations. Although children included in this study met the DDS criteria for autism, they were not systematically evaluated using a common protocol. There was likely a modest level of under-ascertainment of both autism (children with autism who did not seek services in DDS were missed) and ART (ART conceptions out of state or at a non-reporting ART clinic were missed). However, neither of these issues is estimated to have had a major impact on the study population (Croen et al. 2002a; CDC et al. 2012). The children in this study were assessed for autism during the time that the DSM-IV-TR was in place; however, some children in the youngest birth cohorts might have been assessed during the transition to DSM 5. Nonetheless, given the children in our youngest birth cohort (2006) had both mean and median autism diagnosis ages of less than 4 years, we believe the DSM 5, which was published in 2013 when these children were 7 years of age, had minimal impact on autism identification. Our assessment of social and communication severity indicators was hampered by changes in the criteria used during the time period for this study. While we had consistent reporting of co-occurring ID during the entire time frame, IQ scores are not uniformly reported in the DDS for children served under the autism eligibility criterion. Moreover, previous reliability studies (Croen et al. 2002b) and the low overall prevalence of co-occurring ID reported here in comparison to other US surveillance reports (Autism and Developmental Monitoring Network Surveillance Year 2010 Principal Investigators 2014; Autism and Developmental Monitoring Network Surveillance Year 2008 Principal Investigators 2012) are suggestive of under-reporting. While we lacked data to determine if such under-reporting is differential by ART status, it is encouraging that our results point to convergent validity for our various severity indicators; the findings from the communication and social functioning severity indicators matched well with each other and with the findings for co-occurring ID. We were also not able to assess communication and social functioning indicators in the restricted, singleton sample because of sample size constraints. However, given the effects observed in the total sample were already notably attenuated after control for socio-demographics, additional restriction on and control for perinatal risk factors were unlikely to have additional impacts. We were only able to assess the most intensive fertility treatments, those classified as ART, in this analysis. However, conception with non-ART ovarian stimulation treatments has been estimated to be four times as common as ART (Schieve et al. 2009), and children conceived with these treatments face similar increases in adverse perinatal outcomes as ART-conceived children (Schieve et al. 2009; Ombelet et al. 2006) and might also face increased risk for autism (Hvidtjorn et al. 2011). We also lacked data to fully evaluate autism symptomatology in terms of co-occurring disorders such as mood and anxiety disorders. Finally, we were not able to account for the possibility that the children included in our study samples were not completely independent. Given the wide time frame included in this study, it is possible some sibling sets were included; this includes some sets from multiple-birth deliveries in our first two analytic samples.

This study also has a number of strengths. This is one of the largest available samples of children with data on both ART use and autism. The study is population-based and the linkage rates for the various population-based datasets were high. Data were available for a number of important socio-demographic confounding factors as well as for perinatal outcomes found previously to be associated with both ART and autism in US populations (Schieve et al. 2007; Croen et al. 2002b; Durkin et al. 2008; Durkin et al. 2010; Bilder et al. 2009; Mandell et al. 2009; Schieve et al. 2012a). Thus, we were able to thoroughly explore the underlying reasons for the initial differences observed between ART-and non-ART-conceived children.

Children conceived with ART are identified as having autism earlier and are more likely to present with less severe symptomatology than children from the general population. However, after adjustment for the differences in the socio-demographic profiles of the two groups, the diagnosis age differentials were greatly attenuated and there were no differences in autism symptomatology indicating that ascertainment issues related to SES, not ART per se, are likely the driving influence. These findings shed light on one aspect of the emerging data from several studies that report that overall, children conceived with ART are more likely to subsequently be identified as having autism. These findings also have broader implications for understanding ASD prevalence, as they document that ascertainment is quite variable across population subgroups and these differentials may have changed over time.
